# Prevention of Preharvest Sprouting through Hormone Engineering and Germination Recovery by Chemical Biology

**DOI:** 10.3389/fpls.2017.00090

**Published:** 2017-01-31

**Authors:** Mariko Nonogaki, Hiroyuki Nonogaki

**Affiliations:** Department of Horticulture, Oregon State University, CorvallisOR, USA

**Keywords:** chemical biology, germination, hormone, hyperdormancy, inducible gene expression, positive feedback

## Abstract

Vivipary, germination of seeds on the maternal plant, is observed in nature and provides ecological advantages in certain wild species, such as mangroves. However, precocious seed germination in agricultural species, such as preharvest sprouting (PHS) in cereals, is a serious issue for food security. PHS reduces grain quality and causes economical losses to farmers. PHS can be prevented by translating the basic knowledge of hormone biology in seeds into technologies. Biosynthesis of abscisic acid (ABA), which is an essential hormone for seed dormancy, can be engineered to enhance dormancy and prevent PHS. Enhancing nine-*cis*-epoxycarotenoid dioxygenase (NCED), a rate-limiting enzyme of ABA biosynthesis, through a chemically induced gene expression system, has successfully been used to suppress germination of Arabidopsis seeds. The more advanced system *NCED* positive-feedback system, which amplifies ABA biosynthesis in a seed-specific manner without chemical induction, has also been developed. The proofs of concept established in the model species are now ready to be applied to crops. A potential problem is recovery of germination from hyperdormant crop grains. Hyperdormancy induced by the *NCED* systems can be reversed by inducing counteracting genes, such as *NCED* RNA interference or gibberellin (GA) biosynthesis genes. Alternatively, seed sensitivity to ABA can be modified to rescue germination using the knowledge of chemical biology. ABA antagonists, which were developed recently, have great potential to recover germination from the hyperdormant seeds. Combination of the dormancy-imposing and -releasing approaches will establish a comprehensive technology for PHS prevention and germination recovery.

## Introduction

Seed development and germination are not necessarily separate developmental programs in terms of timing (before or after seed dispersal) or location (on the maternal plant or soil). In some species, such as mangroves, these two developmental programs are observed continuously without a temporal or spatial intermission, which is referred to as vivipary (Bewley et al., 2013). However, in many other species, maturation drying, which is natural desiccation of seeds at the late developmental stage, interferes with continuous development of seeds into seedlings. There is a more active mechanism, which suspends germination of developing seeds, that is, seed dormancy. Dormant seeds do not germinate even in the presence of water and under the conditions that are otherwise favorable for germination (Bewley et al., 2013).

Seed dormancy can be found in many wild species while agricultural crops tend to exhibit weak or little dormancy. Seed dormancy traits were present in the wild ancestors of agricultural crops, however, they have been lost over the course of domestication (Nakamura et al., 2016; Subburaj et al., 2016). The lack of dormancy could cause preharvest sprouting (PHS) from grains on the maternal plants in the fields when they are exposed to rain or high humidity.

Genetic research has identified genes associated with seed dormancy and PHS resistance, some of which are associated with grain color (Lin et al., 2016) while others are independent of that trait (Fakthongphan et al., 2016). The genes which play a fundamental role in cellular responses, such as mitogen-activated protein kinase kinase 3 (MKK3), have been identified as a causal gene of PHS in barley (*Hordeum vulgare*) (Nakamura et al., 2016) and wheat (*Triticum aestivum*) (Torada et al., 2016). Interestingly, ARGONAUTE4_9, a key regulator of the RNA-dependent DNA methylation (RdDM) pathway and epigenetic regulation, has also been suggested as a causal gene of PHS in barley (Singh and Singh, 2012) and wheat (Singh et al., 2013).

A variety of genes with different biochemical functions have been identified for PHS resistance or susceptibility. Many of them are associated with hormone signaling, particularly abscisic acid (ABA) signaling (Gao et al., 2012; Gao and Ayele, 2014). It is possible that MKK3 is also associated with ABA signal transduction and/or downstream. The wheat seed dormancy quantitative trait locus (QTL) *QPhs.ocs-3A.1* was found to be *MOTHER OF FT AND TFL1* (*MFT*), a key regulator of ABA signal transduction (Nakamura et al., 2011). An independent analysis of the wheat QTL *Qphs.pseru-3AS* also identified *MFT* as a PHS regulator (Liu et al., 2013). *MFT* has been used as a DNA marker for deep dormancy in wheat (Chono et al., 2015).

The potential of *MFT*, which was originally identified and characterized in the model plant Arabidopsis, for PHS prevention in wheat, suggests that the knowledge of ABA signal transduction obtained from the model plant can directly be translated into cereal crops. ABA insensitive mutations reduce seed dormancy also in wheat (Schramm et al., 2012). Therefore, wheat orthologs of the Arabidopsis genes, which causes ABA hypersensitivity, could enhance seed dormancy and confer PHS resistance to cereals. In fact, mutations in wheat *ENHANCED RESPONSE to ABA* (*ERA*) caused deep dormancy in wheat (Schramm et al., 2013; Martinez et al., 2014) and offer great potential for PHS prevention. Other genes involved in PHS through ABA signaling include ABA-induced *Plasma Membrane-associated protein 19* (*PM19*). *PM19* was suggested to be a seed dormancy regulator in barley (Ranford et al., 2002) and was identified as a dormancy QTL in wheat (Barrero et al., 2015).

Abscisic acid sensitivity has been considered the most critical factor for germination of mature wheat grains. However, there is evidence that ABA metabolism also significantly affect PHS susceptibility and resistance. The importance of ABA 8′-hydroxylase, an ABA deactivation enzyme, in the regulation of barley seed dormancy has been well established (Barrero et al., 2009), which is subject to the regulation by blue light through the CRYPTOCHROME (CRY1) receptor. Blue light inhibits barley seed germination by downregulating *ABA 8′-hydroxylase* and upregulating *nine-cis-epoxycarotenoid dioxygenase* (*NCED*), an ABA biosynthesis gene (Barrero et al., 2014). Wheat ABA biosynthesis and deactivation genes have also been characterized for their function in seed dormancy (Son et al., 2016). Mutations in ABA deactivation genes increased ABA levels in wheat grains and reduced germination (Chono et al., 2013), demonstrating the utility of this approach for PHS prevention. In *Sorghum bicolor, GA2ox*, a gibberellin deactivation gene, plays a critical role in PHS, although this gene is regulated also by the ABA pathway through sorghum ABI4 and ABI5 (Cantoro et al., 2013). These findings demonstrate the robustness of hormone metabolism in the regulation of seed dormancy and germination, which makes engineering of the hormone metabolism pathways in seeds as a logical target of modification in technology development for PHS prevention.

## Switching Off Germination in the Field

The recent advances of hormone biology in seeds provide an excellent foundation for translational biology to prevent PHS. Many genes associated with hormone metabolism in seeds are regulated at the transcriptional level while posttranslational modifications are more prevalent for hormone signal transduction. It is probably more straightforward to alter hormone metabolism through gene expression control, rather than manipulating a certain chemical property (e.g., phosphorylation) of a specific protein involved in ABA signaling.

Enhancing the expression of an ABA biosynthesis gene particularly has great potential to reinforce seed dormancy and prevent PHS. It is possible to spray ABA directly to plants, however, precise control of endogenous ABA is preferable and has been investigated. Expression of *Phaseolus vulgaris NCED* in *Nicotiana tabacum* seeds using the dexamethasone (DEX)-inducible system did not suppress germination adequately (Qin and Zeevaart, 1999). The negative result could be attributed to the experiments performed in the heterologous system or incomplete penetration of the inducible system (Martinez-Andujar et al., 2011). Evidence has been obtained that constitutive expression of *Solanum lycopersicum NCED1* in tomato itself enhances seed dormancy. However, constitutive expression causes undesirable phenotypes in other organs such as leaves (Thompson et al., 2000). It has also been suggested that enhanced levels of ABA could make plants more susceptible to disease (Fan et al., 2009). Therefore, conditional expression of *NCED* in a seed-specific manner is desirable for crops.

Chemically induced gene expression allows conditional expression of *NCED* in seeds, although a steroid hormone agonist like DEX cannot be used for applications in the filed. For agricultural applications, it is necessary to use an inducible system that employs a field-applicable ligand. The Plant Gene Switch System (PGSS), a chemically induced gene expression system (Padidam, 2003; Koo et al., 2004; Tavva et al., 2007), uses methoxyfenozide (MOF), a non-steroid ecdysone agonist, as a ligand. Intrepid2F (Dow AgroSciences), which contains MOF, has been approved by the US Environmental Protection Agency (EPA) and applicable to crop production, making PGSS as a good candidate to be used for PHS prevention. An important question is whether a conditional induction of *NCED*, a single gene, alone would be sufficient to increase ABA levels in seeds and suppress PHS, because many enzymes are associated with the ABA biosynthesis pathway while NCED is believed to be a rate-limiting enzyme (Chernys and Zeevaart, 2000).

**FIGURE 1:**
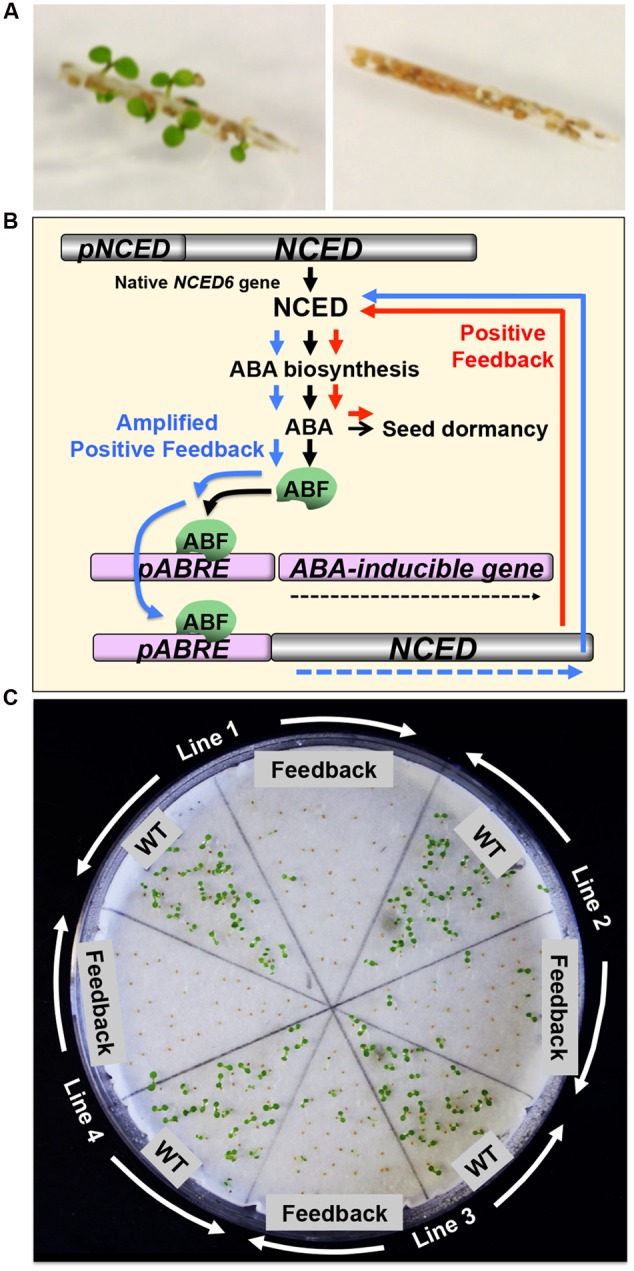
**Preharvest sprouting (PHS) prevention by inducible or spontaneous hyperdormancy through hormone engineering. (A)** Precocious germination of developing Arabidopsis seeds, which was experimentally caused in the silique of the nine-*cis*-epoxycarotenoid dioxygenase (*NCED*)-inducible Plant Gene Switch System (PGSS) line (without *NCED* induction) (*left*). Precocious germination was suppressed by *NCED* induction in the same line (*right*). Modified from Martinez-Andujar et al. (2011). **(B)** Schematic representation of amplified *NCED* expression and enhanced abscisic acid (ABA) biosynthesis and signaling through a positive-feedback mechanism. In the native pathway in seeds (black arrows), *NCED* is expressed to synthesize ABA and induce dormancy. ABA also induces the ABA responsive element (ABRE)-binding factor (ABF), which binds to ABRE in the promoter region of the target genes to induce them. Introduction of the chimeric gene, which contains sorghum *NCED* under the wheat ABA-responsive and seed-specific *Early Methionine-labeled* promoter, into Arabidopsis (bottom), caused positive feedback regulation of *NCED* expression and enhanced dormancy (red arrows), which was further amplified through ABF (blue arrows). See text for details. Modified from Nonogaki et al. (2014). **(C)** Spontaneous hyperdormancy caused by amplified *NCED* expression and enhanced ABA biosynthesis and signaling through a positive-feedback mechanism. Germination of wild-type (WT) and positive-feedback (Feedback) seeds produced from each maternal plant in hemizygous status (Line 1, 2, 3, or 4) is shown. Modified from Nonogaki et al. (2014).

The potential of *NCED* induction by PGSS has been tested in Arabidopsis, which demonstrated that induction of *NCED* alone was robust enough to suspend germination in imbibed seeds. Precocious germination of developing Arabidopsis seeds from the siliques, which was experimentally induced to mimic PHS in cereals, was also prevented by *NCED* induction (Martinez-Andujar et al., 2011) (**Figure 1A**). These results suggest that switching on *NCED* alone is sufficient to switch off germination and maintain seeds dormant. It appears that the ABA biosynthesis pathway upstream of NCED is always running in seeds and constantly providing substrates for NCED. Thus, it is feasible to switch off germination of developing grains in the field and prevent PHS by ligand application if the *NCED*-inducible PGSS is introduced to cereal crops.

## Spontaneous Hyperdormancy in Developing Seeds

While a proof of concept has been obtained for the *NCED*-inducible system with an EPA-approved chemical (Martinez-Andujar et al., 2011), it is laborious to spray the ligand in large fields, which might also affect plant organs other than seeds at the last stage of crop production. It is preferable if *NCED* expression is enhanced specifically in seeds and at the maturation stage without chemical application.

A system that causes ABA increase specifically in maturing seeds in a spontaneous manner, without chemical application, has been sought for. To establish such system, *NCED* can be driven by a seed maturation-specific promoter. However, tissue- or organ-specific and stage-specific promoters may not be as robust as a constitutive promoter. A substantial level of *NCED* expression has to be reached in seeds so that ABA levels will exceed the threshold necessary for dormancy imposition. An idea to address this issue is to use an ABA-responsive promoter to drive *NCED*, which is expected to create a positive-feedback loop through *NCED* expression (**Figure 1B**). An initial increase of ABA caused by the native system in seeds will be enhanced by a positive-feedback mechanism, which will then stimulate the ABA-responsive promoter through activation of the ABA responsive element (ABRE)-binding factor (ABF). The consequence of each round of positive feedback is the further enhancement of *NCED* expression by ABF. Therefore, this mechanism is expected to amplify ABA production to an unusually high level in seeds and cause hyperdormancy spontaneously. Since this “ABA-responsive ABA biosynthesis” occurs in the only seed tissues that produces native ABA at the right timing, the positive-feedback system is devoid of pleiotropic effects.

This idea has been tested using the *Triticum aestivum Early Methionine-labeled* (*TaEM*) promoter, which is an ABA-responsive seed maturation-specific promoter, and *Sorghum bicolor NCED* (*SbNCED*) (Nonogaki et al., 2014). Introduction of this system (*pTaEM:SbNCED*) into Arabidopsis increased ABA levels in seeds up to ∼70-fold compared to those in wild-type (WT) seeds and caused unusually deep hyperdormancy (**Figure 1C**). These results suggest that the sorghum NCED functioned efficiently in the metabolic pathway in Arabidopsis and the Arabidopsis transcription factors were able to activate the wheat promoter. The basic mechanisms of hormonal regulation of seed dormancy seem to be conserved between monocot and dicot species. Since the chimeric gene was constructed using the cereal promoter and coding gene and functioned in Arabidopsis properly, it is anticipated to function equally in cereal crops if not better. The *NCED* positive-feedback system is currently introduced to cereal crops for further investigation.

## Switching on Germination After Seed Harvest

Hyperdormancy is desirable for PHS prevention during crop production. However, the extremely deep dormancy maintained in harvested grains could be problematic when they are used as seeds and need to germinate for the next round of crop production. It is essential to secure strategies for seed germination recovery from the PHS-resistant hyperdormant seeds. To this end, PGSS can be used to induce positive regulators of seed germination, such as GA biosynthesis genes. While ligand application in large fields during crop production may not be practical (see above), it is highly feasible to apply a chemical ligand to harvested seeds in a warehouse. Many seed companies use wet treatments for vegetable and flower seeds. Cereal grains, as starting materials for crop production, can also be treated by chemicals in a small scale. Thus, PGSS offers a suitable method for seed germination recovery if the induction of counteracting gene(s) is sufficient to reverse the suppression of germination caused by *NCED* expression.

A potential problem is the permeability of seed covering tissues, such as the testa and pericarps. In the basic experiments in Arabidopsis, the chemical ligand was able to reach the endosperm (and most likely through the embryo also) after testa rupture (Martinez-Andujar et al., 2011). However, testa rupture does not occur in dormant seeds, including cereal grains. If the pericarp and testa of cereal grains are impermeable to the ligand, a sufficient level of gene induction may not occur, which hinders dormancy release and germination recovery. Therefore, an inducible gene expression system that employs a testa-permeable chemical ligand needs to be developed for efficient recovery of seed germination from hyperdormant seeds.

**FIGURE 2:**
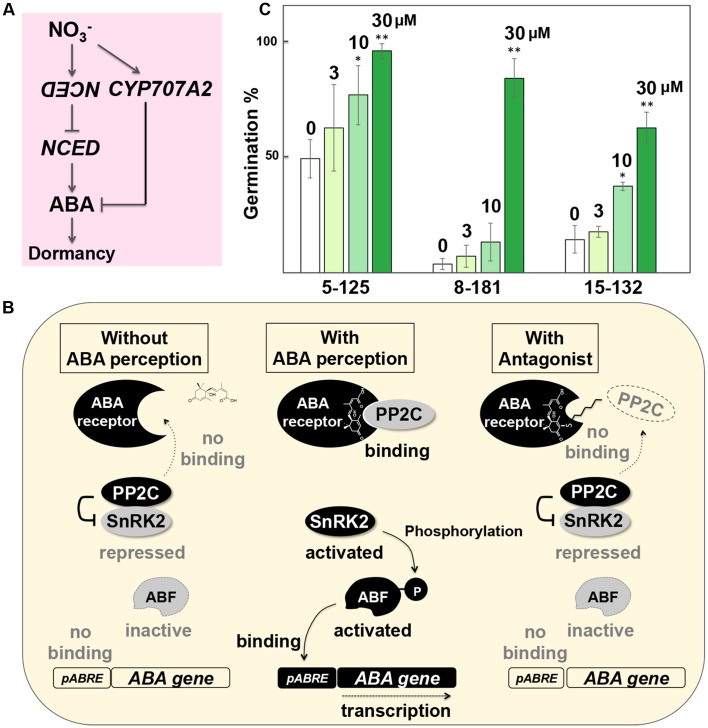
**Recovery of germination from PHS-resistant hyperdormant seeds. (A)** Schematic representation illustrating the dual effects of antisense *NCED* or *NCED RNAi* induction in seeds by a nitrate-inducible gene expression system. Induction of anti-*NCED* genes using a nitrate-responsive promoter could counteract with enhanced *NCED* expression in the inducible or spontaneous hyperdormant seeds. At the same time, nitrate induces *CYP707A2*, an ABA deactivation gene, in the native system in seeds, which also reduces ABA. Therefore, nitrate-inducible anti-*NCED* gene expression could have dual effects to break dormancy and recover germination. **(B)** Schematic representation of germination recovery by a chemical biology approach. In the absence of ABA (*left*), protein phosphatase 2C (PP2C) suppresses the downstream events necessary for ABA signal transduction by binding to SNF1-related protein kinase 2 (SnRK2). In hyperdormant seeds (*middle*), ABA triggers PP2C binding to the receptor and causes ABA signal transduction, which suppresses germination. Application of 3′-hexylsulfanyl-ABA (AS6), an ABA antagonist (*right*), blocks PP2C binding due to the protruded *S*-hexyl chain and hinders ABA signaling, thereby making seeds insensitive to ABA. Based on Hayashi and Kinoshita (2014) and Takeuchi et al. (2014). **(C)** Recovery of germination by 3′-hexylsulfanyl-ABA (AS6), an ABA antagonist, from the *NCED*-induced hyperdormant seeds. Seeds of the three independent *NCED*-inducible lines (5-125, 8-181, 15-132) were treated with the chemical ligand Intrepid2F to suppress germination (0 μM [AS6]), from which germination was recovered by AS6 in a dose (3, 10, 30 μM)-dependent manner. ^∗^*P* < 0.05, ^∗∗^*P* < 0.01 (Student’s *t*-test compared with 0 μM).

Nitrate permeates through the testa and could serve as an efficient inducer of gene expression. The nitrate responsive *cis*-element (NRE) in the promoter region of *NITRITE REDUCTASE1* (*NIR1*), which is involved in nitrate-responsive gene expression, has been characterized (Konishi and Yanagisawa, 2010). Induction of a counteracting gene, such as anti-*NCED* or *NCED* RNA interference (RNAi), using the nitrate-inducible system, is expected to antagonize the enhanced *NCED* expression in the hyperdormant seeds and reduce ABA levels in seeds (**Figure 2A**). There is another advantage of using the nitrate-inducible gene expression system for seed germination recovery. Nitrate activates nodule inception (NIN)-like protein 8 (NLP8), which directly binds to the promoter of *CYP707A2*, an ABA deactivation gene, and releases seed dormancy in the native system of Arabidopsis seeds (Yan et al., 2016). Therefore, nitrate application could have dual effects of reducing ABA biosynthesis (*NCED* expression) and enhancing ABA deactivation (*CYP707A2*), both of which reduce ABA levels and promote germination (**Figure 2A**). Nitrate-inducible expression of germination-promoting genes using the *NIR1* promoter has not been tested yet. However, the potential of this promoter for gene induction in seeds has already been tested, which was demonstrated to be efficient for the induction of a test gene (long non-coding RNA) in seeds at the stage before testa rupture (Nonogaki et al., 2015). Thus, nitrate has potential to induce gene expression in seeds at the early imbibitional stages and switch on germination in PHS-resistant seeds.

## Chemical Biology to Recover Germination

It is possible that induction of counteracting genes by PGSS is still insufficient to reduce (in the case of anti-*NCED*) or antagonize (in the case of *GA3ox*) ABA levels in the PHS-resistant seeds, which prevents germination. A strategy to overcome this issue is to alter the sensitivity of seeds to ABA. Even when ABA levels in seeds are still higher than the threshold to maintain dormancy, if the ABA sensitivity of seeds is reduced, the biochemical events that are necessary to alleviate dormancy but are blocked by ABA signaling may be allowed to happen, which should results in seed germination.

Abscisic acid signaling is initiated by perception of ABA by its receptor, to which protein phosphatase 2C (PP2C) binds. As a consequence, SNF1-related protein kinase 2 (SnRK2), which was suppressed by PP2C, is activated and phosphorylates the downstream factor ABF (Cutler et al., 2010). ABF then binds to ABRE to induce downstream genes (**Figure 2B**). Since these sequential events in ABA signal transduction depends on PP2C binding to the receptor, if the initial interaction is blocked, ABA signaling can be inhibited efficiently. Based on this concept, ABA antagonists, which are capable of binding to the receptor but prevent its interaction with PP2C, have been developed. For instance, 3′-hexylsulfanyl-ABA (AS6) blocks PP2C binding to its receptor due to the protruded *S*-hexyl chain and hinders ABA signaling (Hayashi and Kinoshita, 2014; Takeuchi et al., 2014) (**Figure 2B**).

Although imbibed hyperdormant seeds maintain ABA biosynthesis at unusual levels (Nonogaki et al., 2014), if seeds become insensitive to ABA temporarily during early imbibition due to the presence of an ABA antagonist, they are expected to germinate. Application of AS6 has been tested with the *NCED*-inducible hyperdormant seeds. While the effects of AS6 differ among independent lines, AS6 application recovers germination from the *NCED*-induced seeds in a dose-dependent manner (**Figure 2C**), supporting the idea of seed germination recovery from PHS-resistant seeds by a chemical biology approach. There are occasions in which AS6 is not potent enough to affect dormant seeds, such as the WT accession of Arabidopsis seeds Cape Verde Islands (Cvi) (Rajagopalan et al., 2016). However, new antagonists, which are more potent than AS6 in terms of dormancy release, have also been developed recently (Takeuchi et al., 2015; Rajagopalan et al., 2016). It is possible that some antagonists do not permeate the testa and pericarp efficiently. Nonetheless, this issue also can be addressed by exploring and modifying ABA antagonists with different chemical properties. Thus, chemical biology also offers new strategies for seed germination recovery from PHS-resistant seeds.

## Conclusion

Combination of hyperdormancy and seed germination recovery strategies will potentially establish a comprehensive technology for PHS prevention. Although this Perspective focused on hormone engineering, other strategies, such as manipulation of redox proteins, could also enhance seed dormancy and delay PHS (Li et al., 2009). In any case, all of these approaches require genetic engineering of cereal crops, which is subject to regulatory processes. The mutant and other genetic lines mentioned in Introduction can be used to confer PHS resistance to commercial varieties through traditional breeding. However, introduction of those genes and traits into the major varieties through crossings and selections will take a long time. Besides, the same varieties will need to go through another breeding program to transfer a seed germination recovery strategy, which will make further delays. Climate changes could cause unexpected and serious problems of PHS in various grain crops, which are food security issues. It is crucial to confer PHS resistance to the major crops used in global production. A possible game changer is the new technologies of crop modification, such as gene editing, which does not leave engineering tools in the final products and may not be subject to the regulations (Ledford, 2015; Waltz, 2016). These new methodologies could accelerate technology development for PHS prevention. Whether through genetic engineering or traditional breeding, progressive ideas to prevent PHS and recover germination need to be tested in the major crops so that new technologies will be established and can be utilized immediately when PHS issues become even more serious.

## Author Contributions

MN and HN designed and performed the research, analyzed the data, and wrote the paper.

## Conflict of Interest Statement

A patent application has been filed for a technology described in this article.
